# CXCR2 blockade potentiates HDAC3 deletion mediated antitumor immunity in colorectal cancer

**DOI:** 10.1038/s41419-026-09006-3

**Published:** 2026-07-10

**Authors:** Yukun Zhang, Yeping Yu, Fang Yang, ZiHeng Zhao, Xiaodong Gu, Zhenyang Li, Yiming Zhou, Wenqian Zhang, Huichun Chen, Jianbin Xiang

**Affiliations:** 1 Department of General Surgery, Huashan Hospital, Fudan University, Shanghai, China; 2 Shanghai Institute of Immunology & Department of Immunology and Microbiology, Key Laboratory of Cell Differentiation and Apoptosis of Chinese Ministry of Education, Faculty of Basic Medicine, Shanghai Jiao Tong University School of Medicine, Shanghai, China; 3 Department of General Surgery, The Third Affiliated Hospital of Wenzhou Medical University, Wenzhou, Zhejiang China

**Keywords:** Cancer microenvironment, Immune evasion, Immunoediting

## Abstract

HDAC3 is highly expressed in colorectal cancer (CRC) and associated with poor prognosis. This study reveals that HDAC3 deletion dually regulates chemokine expression through epigenetic mechanisms, promoting CD8⁺ T cell infiltration (via CXCL10) on one hand, while enhancing the recruitment of myeloid-derived suppressor cells (MDSCs) (via CXCL1/2/3/5) on the other. The latter, mediated through CXCR2 signaling, counteracts the intrinsic anti-tumor effect of HDAC3 deletion. We further demonstrate that combining a CXCR2 inhibitor effectively blocks MDSC infiltration and significantly enhances the immunotherapeutic efficacy mediated by HDAC3 deletion. This study systematically elucidates the “double-edged sword” role of HDAC3 in the CRC immune microenvironment and provides a theoretical and experimental basis for an HDAC3/CXCR2 dual-targeting therapeutic strategy.

## Introduction

Colorectal cancer (CRC) is the third most common malignancy worldwide [1]. Recent advances in the molecular and genetic understanding of CRC biology, along with the development of targeted therapy and immunotherapy, have significantly altered treatment strategies for CRC patients [2]. Dysregulation of epigenetic regulators has been implicated in the pathogenesis of human CRC, and targeting these regulators has emerged as a promising therapeutic approach for CRC treatment [3].

Histone deacetylases (HDACs) are a family of epigenetic enzymes that suppress gene expression by removing acetyl groups from lysine residues on histone proteins [4, 5]. HDACs influence a variety of cellular processes, including differentiation, cell cycle progression, and apoptosis [6]. Aberrant HDAC expression has been implicated in the development of multiple human cancers. Pan- and selective HDAC inhibitors have shown potential to suppress tumor growth by inducing cell-cycle arrest and apoptosis, and are already being used in the treatment of certain hematologic malignancies [7, 8]. Beyond their direct cytotoxic effects, HDAC inhibitors can modulate the tumor immune landscape by altering the expression of costimulatory and coinhibitory molecules, MHC molecules, tumor antigens, and cytokines [9–11]. These inhibitors can also impact the phenotypes of immune cell subsets within the tumor immune microenvironment (TIME) [5, 12, 13]. Recent studies have also demonstrated that tumor-specific HDAC deletion can up-regulate chemokine expression, thereby influencing immune cell infiltration into the TIME and shaping antitumor immunity [14–16].

Various HDAC isoforms exhibit distinct subcellular localizations and target proteins, which contribute to their diverse biological functions [17, 18]. Among these, HDAC3 is deregulated in CRC and serves as both a complementary molecular marker for histopathological diagnosis and a prognostic biomarker. HDAC3 has been implicated in CRC progression and metastasis, with overexpression shown to promote cell proliferation and invasion [19, 20]. However, a recent study reported that HDAC3 deficiency does not affect fibrosarcoma cell growth or survival. In contrast, tumor-specific deletion of HDAC3 enhances antitumor immunity in a murine fibrosarcoma model by increasing CD8^+^ T cell infiltration into the TIME via upregulation of CXCL9/10/11 [14]. In T cell lymphoma, HDAC3 inhibition has been shown to upregulate CXCL12 secretion by tumor cells, thereby recruiting NK cells into the TIME [15]. These findings suggest that targeting HDAC3 in tumor cells could facilitate the recruitment of immune cells with antitumor activity. Immunosuppressive cells, including M2 macrophages, myeloid-derived suppressor cell (MDSC), and regulatory T cells, exist in the TIME and play a crucial role in immune escape, thereby affecting the immunotherapy effect [21–23]. It remains unclear whether tumor-specific inhibition of HDAC3 would also increase the infiltration of other immunosuppressive cells within the TIME, leading to impairment of its antitumor immunity. Clinical data provide some insight: a study (CAPability-01) combining HDAC1/2/3 inhibitors (chidamide) with anti-PD-1 (sintilimab) and anti-VEGF (bevacizumab) showed promising results in patients with unresectable, chemotherapy-resistant, locally advanced or metastatic microsatellite-stable (MSS) CRC [24]. Notably, sequencing data from post-treatment tumor tissues revealed that, in patients with significant tumor regression, CD8^+^T cell infiltration was markedly increased, whereas in patients with disease progression and treatment resistance, neutrophil infiltration was elevated, and CD8^+^ T cell infiltration was reduced [24]. These observations suggest that HDAC3 inhibition may exert context-dependent effects on tumor immunity, promoting either antitumor or pro-tumor immune responses [16].

Therefore, to systematically elucidate this “double-edged sword” role of HDAC3 in CRC immune regulation, we conducted an integrated study starting from clinical samples, employing multi-omics analysis, gene editing, and functional assays both in vivo and in vitro. Our study unveils that HDAC3 deletion dually regulates chemokine expression, specifically enhancing both CD8⁺T cell-attracting CXCL10 and MDSC-recruiting CXCL1/2/3/5 through epigenetic reprogramming. Furthermore, we propose and validate a viable combination therapy strategy by concurrently blocking the CXCR2 pathway to unleash the full antitumor potential of HDAC3 inhibition.

## Materials and methods

### Clinical CRC specimens

Human CRC samples were obtained from the patients who were diagnosed with CRC by pathological examination in the Huashan Hospital, Fudan university. This study was approved by the Institutional Research Ethics Committee of Huashan Hospital, Fudan university, and written informed consent was obtained from all patients.

### Cell lines and cell culture

CRC cell lines (MC-38, CT-26, HCT-116 and SW-620) were originally from American Type Culture Collection. MC-38 and CT-26 cells were cultured in RPMI 1640 media with 10% Fetal Bovine Serum and 1% penicillin-streptomycin at 37 °C under 5% CO_2_. HCT-116 and SW-620 were cultured in McCoy’s 5A modified medium and Dulbecco’s modified Eagle medium (DMEM), respectively. All cell lines were examined without mycoplasma contamination.

### CRISPR-Cas9 knockout of HDAC3

Single guide RNAs (sgRNAs) targeting the murine and human HDAC3 gene were designed on the Synthego website (https://www.synthego.com) and cloned into lentiCRISPR v2 (Addgene, Cambridge, MA). sgRNA sequences for HDAC3 were listed in Supplementary Table. For lentivirus production, 3 μg of plasmid DNA, 2.25 μg of psPAX2 (Addgene #12260), 0.75 μg of pMD2.G (Addgene #12259) was mixed with P3000 in Opti-MEM, and lipofectamine 3000 (Invitrogen) was mixed with Opti-MEM in another tube. After 15 min, contents of the two tubes were mixed and added to HEK293T cells cultured in a 6-cm cell dish. The medium was changed with complete cell culture medium after 6 h of transfection, and the lentivirus supernatant was collected after 48 and 72 h. To get HDAC3 knockout cell lines, 5 × 10^5^ cells were infected with lentivirus supernatant for 48 h. After 7 days’ puromycin treatment, single cell clones were selected and confirmed by western blot.

### Western blot

Cells were lysed with RIPA (Beyotime, #P0013B) containing protease inhibitors and phosphatase inhibitors. The protein concentration was measured by BCA assay (Thermo Fisher, #23227). For western blot, 30 µg protein was loaded into 10% SDS-PAGE gel, and then transferred to a PVDF membrane. After blocking with 5% non-fat milk in TBST, the PVDF membrane was incubated with primary antibody overnight at 4 °C, after washed three times with TBST, the PVDF membrane was incubated with secondary antibody for one hour at room temperature. Then, the PVDF membrane was washed three times with TBST, and visualized by ImageQuant LAS 3000 instrument (GE Healthcare, Little Chalfont). The information of western blot antibodies was listed in Supplementary Table. Western blot results were quantitatively analyzed by ImageJ (v1.8.0).

### Cell proliferation assay

CRC cell lines were cultured in 96-well plates at a density of 3000 cells in 100 μl medium per well. After incubated at 37 °C for 48 h, the medium of CRC cells was changed with CCK8 containing medium (10  μl CCK8 in 90  μl culture medium) and further incubated at 37 °C for 30 min to 4 h. Finally, the absorbance was measured at a wavelength of 450 nm.

### Apoptosis assay

After incubated at 37 °C for 48 h, the HDAC3-KO or RGFP966/JQ1 treated CRC cells were collected and stained with Annexin V and PI (Thermal Fisher, #BMS500FI-300) according to the manufactures’ instructions. The cells were tested on FACS LSR Fortessa X20, and the data were analyzed by FlowJo software (FlowJo v10).

### RNA-seq and data analysis

Total RNA of 1 × 10^7^ CRC tumor cells was extracted by Trizol Reagent according to the manufacturer’s instructions. RNA quality and concentration was examined by 5300 Bioanalyser (Agilent) and NanoDrop-2000 (NanoDrop Technologies). RNA libraries were sequenced on a Nextseq500 sequencer according to manufacturer’s instructions. Then clean reads were separately aligned to reference genome by HISAT2 software. The mapped reads of each sample were assembled by StringTie. Differential expression analysis between two groups of different samples was performed by R package DESeq2. GO functional-enrichment analysis was analyzed by Gorilla. GSEA software (v.4.0.3) was applied for Gene Set Enrichment Analysis (GSEA). Adjusted *P* value < 0.05 and log2|fold-change|> 1.5 was considered as statistically significant.

### RNA isolation and reverse transcription-quantitative PCR (qRT-PCR)

Total RNA was extracted by TRIzol according to the manufacturer’s instructions. The quality and concentration of RNA was examined by NanoDrop-2000. The cDNA was synthesized by PrimeScript™ RT reagent Kit (Takara, #RR036A) for qPCR. Real-time PCR was performed with HiScript II One Step qRT-PCR SYBR Green Kit (Vazyme) on ViiA 7 Real-Time PCR System (Applied Biosystems). The primer sequences are shown in Supplementary Table. Relative expression changes were calculated by 2^–ΔΔCT^ method.

### Enzyme-linked immunosorbent assay (ELISA)

Mouse CXCL1, CXCL2, CXCL3 and CXCL5 concentrations were quantified using a commercial ELISA kits (Jianglai Biology) according to the manufacturer’s instructions. Briefly, serially diluted supernatants or serum samples were added to pre-coated plates and incubated at 37 °C for 1 h. After washing, biotin-conjugated detection antibodies were applied, followed by streptavidin–HRP. Tetramethylbenzidine substrate was then added, and absorbance at 450 nm was measured using a microplate reader (BioTek Synergy HT).

### Cell isolation and in vitro migration assay

MDSCs or CD8^+^ T cells were isolated from spleens of 8-week-old female C57BL/6 mice by EasySep™ Mouse MDSC/CD8^+^T cell Isolation Kit (STEMCELL Technologies). To perform MDSC/CD8^+^ T cell migration assay in vitro, 5 × 10^4^ MDSCs/CD8^+^ T cells were seeded into upper chambers of 24-well transwell plates (Costar Corning). Conditioned medium from control MC-38 cells or HDAC3 knockout MC-38 cells was filtered and added into the lower chambers. 10 µM SB225002 (Selleck) was added into the upper chambers to block CXCR2. Cells in the lower chambers were counted and analyzed after 4 h.

### The assay for transposase-accessible chromatin with sequencing (ATAC-Seq)

Briefly, MC-38 cells (~50,000 cells per group) were collected and washed by Phosphate Buffered Solution. Cells were lysed and centrifuged for nucleus. DNAs were broken by TN5 Transposase (Vazyme) and purified by VAHTS DNA Clean Beads (Vazyme). DNA was enriched by Polymerase Chain Reaction (PCR) and purified again. Fragment distribution and quality was evaluated by Agilent 2100 Bioanalyzer and libraries sequencing was performed on Illumina Novaseq X plus platform.

### Cleavage under targets and tagmentation (CUT&Tag)

The Hyperactive Universal CUT&Tag Assay Kit for Illumina (Vazyme, cat#TD904) was applied in this experiment. 100,000 events of HDAC3-KO or control MC-38 cells were collected at room temperature and incubated with Concanavalin A-coated Magnetic Beads Pro and anti-H3K27ac antibodies, BRD4 antibodies or control IgG (Cell Signaling Technology) at 4 °C overnight. Next, cells were incubated with corresponding secondary antibody, after combining Hyperactive pA/GTransposon Pro and fragmentation, the DNA was extracted and applied for constructing sequencing library with custom primers. Finally, library DNA was purified by DNA Clean Beads (Vazyme, # N411-01). Eluded DNA was subjected for sequencing or quantitative PCR (qPCR) analysis. The sequence for the primers is provided in the Supplementary Table.

### ATAC-seq and Cut&Tag data analysis

Paired end FASTQ files were trimmed to remove adaptor sequences by “Fastp” (v 0.20.1), and then were aligned to the mm10 mouse genome by Bowtie2 (v 2.4.2) with the“–end-to-end–very-sensitive–no-mixed–no-discordant–phred33-I10-X700” options. High-quality reads were converted to Bigwig files by deepTools (v.3.1.3). Significant peaks were called by MACS2 v2.2.4 with default settings. Next, the peaks were annotated by Chipseeker. Heatmaps were generated by the deepTools function plotHeatmap. And the differential analysis and enrichment analysis were performed on R (v2.4.3). Finally, IGV (Integrative Genomics Viewer, v2.12.3) browser was applied for generating peak figures of CXCL1/2/3/5/10 in two groups.

### Syngeneic mouse tumor models and inhibitor treatment

Female C57BL/6J mice (6–8 weeks old) were subcutaneously injected with MC38-HDAC3-KO or MC38-sgControl cells (5 × 10^5^ cells per mouse). Five days after tumor cell inoculation, mice were randomly allocated to different groups, and administrated with 50 mg SB225002 (Selleck), 50 mg RGFP966 (Selleck) or control solvent every other day via intraperitoneal injection (i.p.). Seven days after tumor cell inoculation, tumor size was measured every three days by a digital calliper, and tumor volume was calculated and record (volume = 0.5 × length × width^2^). Mice were killed to assess the tumor weight and tumor microenvironment at the indicated timepoint. All animal experiments in this study comply with ARRIVE (Animal Research: Reporting of In Vivo Experiments) guidelines and were approved by the Department of Laboratory Animal Science, Fudan university.

### Flow cytometry

For flow cytometry, tumor tissue was digested into single cells and stained with antibodies for 30 min at 4 °C, the antibody panels of various immune cell types are provided in Supplementary Table. Flow cytometry was implemented on FACS LSR Fortessa X20, and data were analyzed by FlowJo software.

### Immunohistochemistry and Immunofluorescence

Tumor tissues were fixed, Paraffin-embedded and sectioned. After deparaffinized, rehydrated and boiled in citrate Buffer for antigen retrieval, sectioned tissues were blocked with 5% BSA, and incubated with primary antibodies at 4 °C overnight in humidified box. The list of antibodies was shown in Supplementary Table. For immunohistochemistry, slides were incubated with HRP-conjugated secondary antibodies and developed with DAB Plus. For immunofluorescence, slides were incubated with fluorescent conjugated anti-CD11b, anti-Gr1 and anti-CD8 antibody, finally stained with DAPI and eventually examined by fluorescence microscope.

### Statistical analysis

All data were presented as mean ± s.d. or mean ± s.m. At least two independent biological replicates were conducted for in vitro assays and animal experiments. Western blot results were quantitatively analyzed by ImageJ (v1.8.0). Statistical analysis was performed using Prism (GraphPad Software, v8.0), and differences between two independent groups were analyzed with Unpaired Student’s *t* test. Comparisons among three or more groups were analyzed by one-way analysis of variance (ANOVA). Two-way ANOVA was applied to compare growth curves. Correlation analysis was conducted using Pearson Correlation test or Spearman Correlation test. *P* value < 0.05 was considered as statistically significant.

## Results

### HDAC3 expression is upregulated in CRC and is associated with low immune activation

**Fig. 1:**
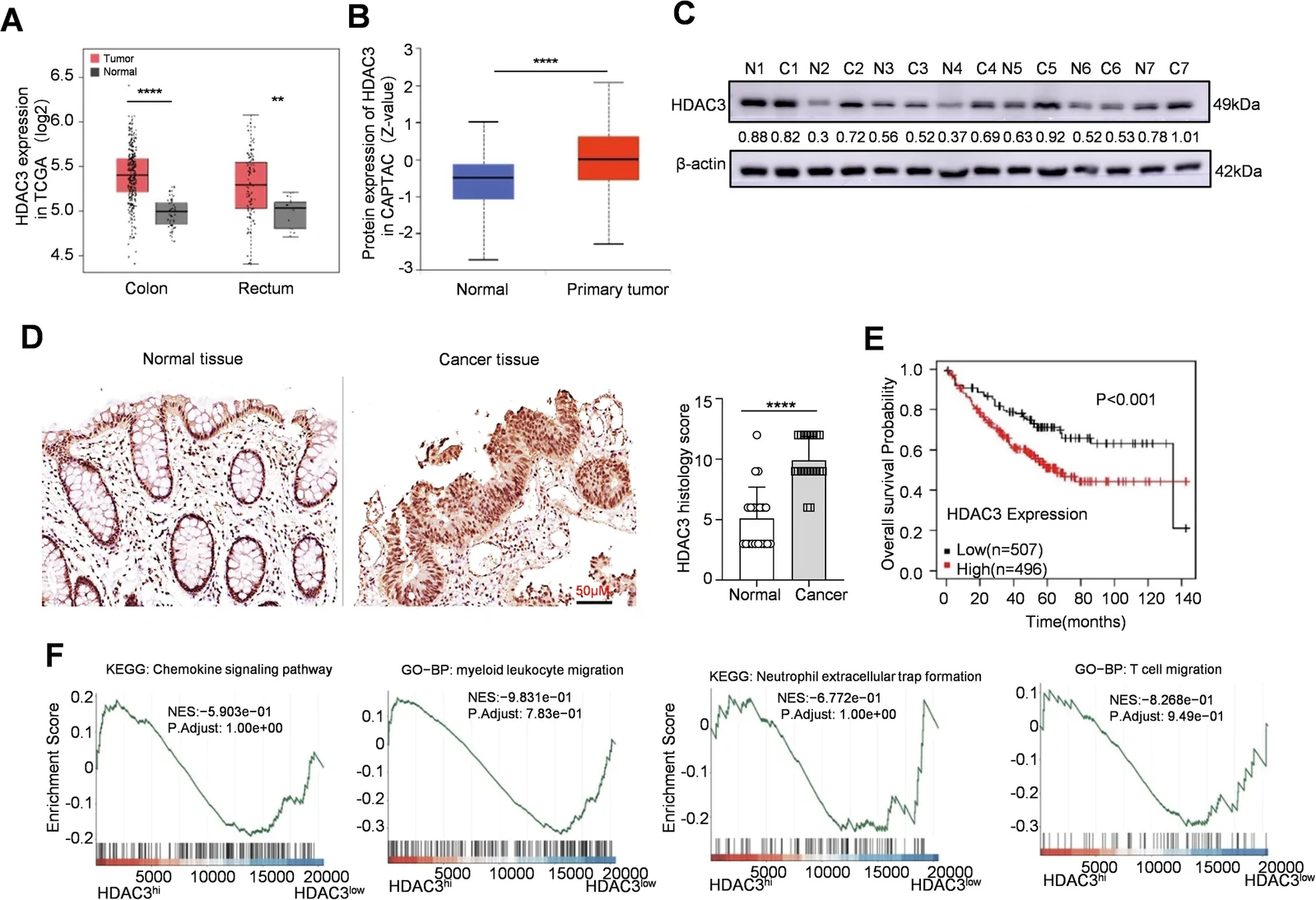
HDAC3 is a crucial pro-oncogenic protein associated with low immune activation in CRC. **A** Boxplots showing the relative HDAC3 mRNA levels in human colon cancer specimens (*n* = 275), rectum cancer specimens (*n* = 92) and adjacent normal tissues (*n* = 41 and *n* = 10) by analyzing the TCGA database. **B** Boxplots showing the relative HDAC3 protein levels in human CRC and matched normal tissues by analyzing the UALCAN database. **C** Western blotting analysis of HDAC3 protein in human CRC tissues (C) and matched adjacent normal tissues (*n* = 6). **D** Representative images of immunohistochemical staining for HDAC3 in CRC tumor tissues and matched adjacent normal tissues (scale bar, 50 μm) and statistical analysis. **E** Kaplan–Meier curves of overall survival (*n* = 1003) in patients from TCGA-COAD dataset stratified by the HDAC3 mRNA levels. **F** GSEA showing the depletion of the indicated pathway signatures in CRC patients with higher HDAC3 expression. Data are shown as mean ± s.d. (**A**, **B**, **D**). Statistical significance was evaluated by one-way ANOVA (**A**, **B**), Student’s *t* test (**D**) and Kolmogorov–Smirnov test (**F**). **P < 0.01 and ****P < 0.0001.

Numerous studies have demonstrated that HDAC3 mRNA and protein expression levels are up-regulated in malignant tumors. In consistent with these reports, we also found that HDAC3 was significantly overexpressed in human CRC tissues compared to adjacent normal tissues at both mRNA and protein levels by analyzing public database (Fig. 1A, B and Supplementary Fig. S1A). Moreover, Western blotting and immunohistochemistry analysis also showed an increase in HDAC3 protein in some human CRC specimens compared to adjacent normal tissues (Fig. 1C, D). Importantly, elevated HDAC3 expression correlates with poor outcomes for patients with CRC (Fig. 1E), even following chemotherapy and targeted therapy (Supplementary Fig. S1B, C). Moreover, CRC with lower HDAC3 levels displayed the enrichment of multiple immune-related pathways such as chemokine signaling (Fig. 1F). These results suggest a potential role of HDAC3 in modulating tumor immunity in CRCs.

### HDAC3 deletion induces chemokine reprogramming

**Fig. 2:**
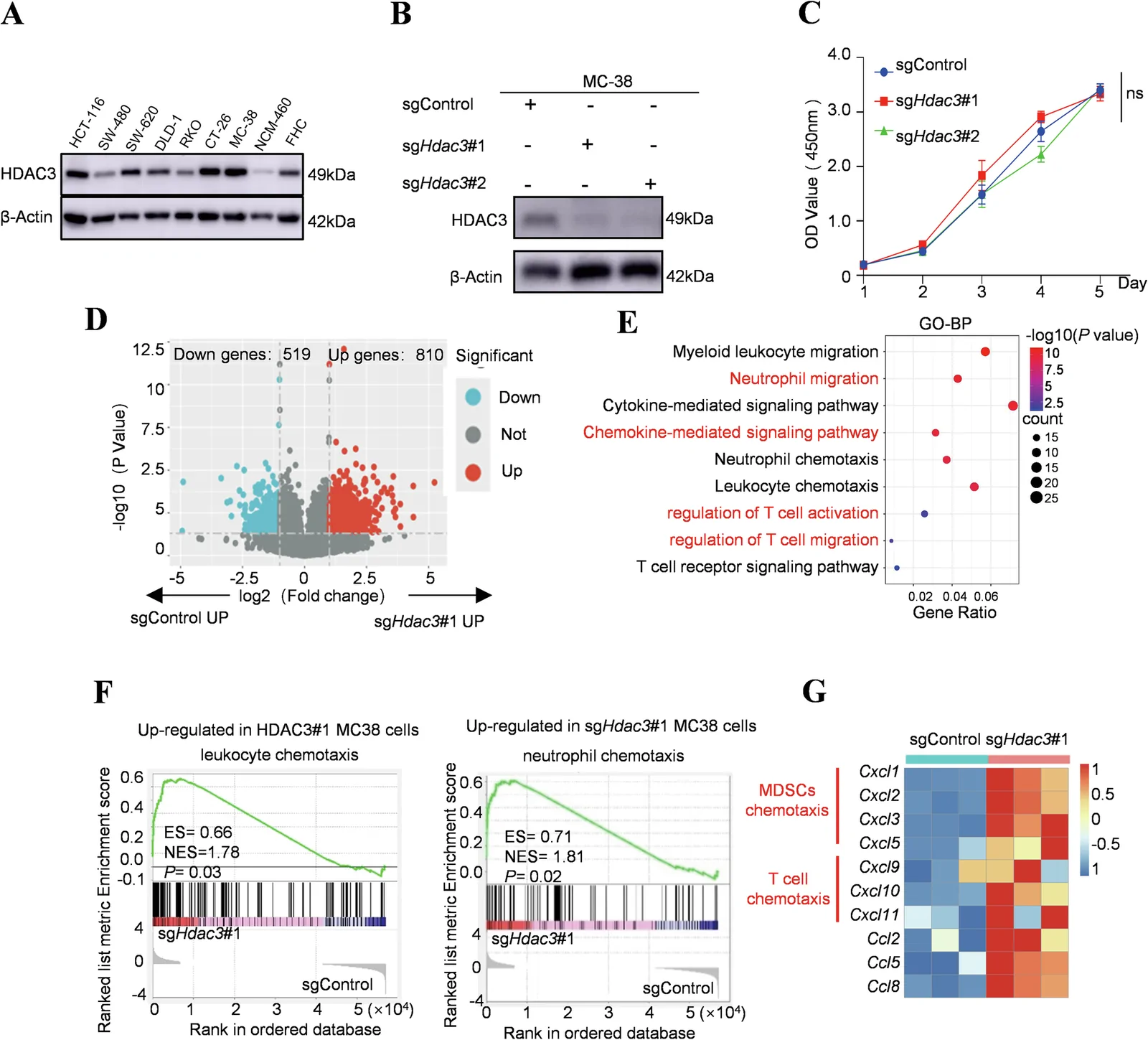
HDAC3 deletion up-regulates the expression of CXCL1/2/3/5/10 in MC38 cells. **A** Western blot analysis of HDAC3 expression in CRC cell lines. **B** Western blotting analysis of HDAC3 protein in indicated gRNA-transduced MC-38 cells. **C** Cell growth curve of *Hdac3*-deficient and control MC-38 cells (*n* = 3). **D** Volcano plots showing differential expression genes in *Hdac3*-deficient and control MC38 cells. **E** GO-BP pathways in up-regulated genes in *Hdac3*-deficient MC38 cells compared to control cells. **F** GSEA enrichment analysis of the neutrophil chemotaxis pathway in *Hdac3*-deficient MC38 cells. **G** Heatmap of differential chemokine-related genes between *Hdac3*-deficient MC38 cells and control cells. Data are shown as mean±s.d. of triplicate experiments. Statistical significance was evaluated by two-tailed two-way ANOVA (**C**). ns not significant.

**Fig. 3:**
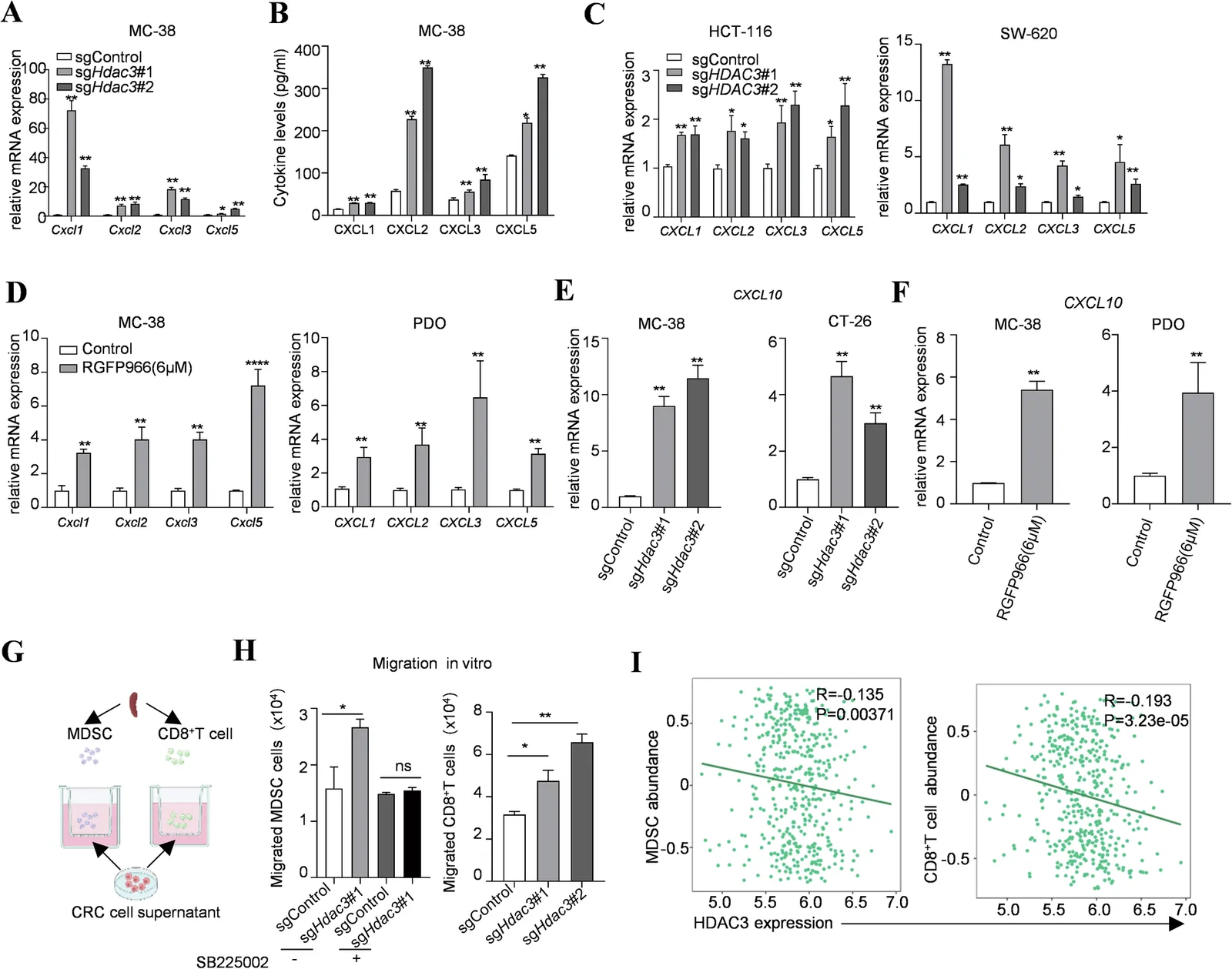
HDAC3 inhibition simultaneously enhances chemotaxis of CRC towards CD8^+^ T cells and MDSCs in vitro. **A** Relative mRNA levels of indicated genes in MC-38 cells, determined by RT-qPCR (*n* = 3). **B** The concentration of indicated chemokine in the supernatant from cultured *Hdac3*-deficient and control cells (*n* = 3). **C** Relative mRNA levels of *CXCL1/2/3/5* mRNA in HCT-116 and SW-620 cells (*n* = 3), determined by qPCR. **D** Relative mRNA levels of *CXCL1/2/3/5* mRNA in MC-38 cells and PDOs after treatment of RGFP966 (*n* = 3), determined by qPCR. **E** Relative *Cxcl10* mRNA levels in sg*Hdac3*-MC-38 or CT-26 cells as determined by RT-qPCR (*n* = 3). **F** Relative *CXCL10* mRNA levels in MC-38 cells or PDOs treated with RGFP966, determined by RT-qPCR (*n* = 3). **G** Workflow of MDSC or CD8^+^ T cell migration assay. **H** Left, Quantification of migration of MDSCs or CD8^+^ T cells toward sgControl or sg*Hdac3*-MC-38 conditioned medium in the presence of SB225002 or not (*n* = 3). **I** Dot scatter plots showing the correlations between the abundance of HDAC3 expression and MDSCs (left) or CD8^+^ T cells (right) in human CRC specimens. *P* value was determined by Pearson correlation test. Data are shown as mean ± s.d. Statistical significance was evaluated by Student’s *t* test (**A**–**F**, **H**). ns not significance; *P < 0.05 and **P < 0.01.

To further explore the influence of HDAC3 on CRC progression, we assessed the level of HDAC3 protein in various CRC cell lines, and CRISPR-Cas9 technology was employed to generate *Hdac3*-deficient MC-38 cells (Fig. 2A, B). Notably, *Hdac3* deletion did not significantly impact cell proliferation and apoptosis of MC38 cells (Fig. 2C and Supplementary Fig. S2A–D), in consistent with a recent study, indicating that *Hdac3* may regulate CRC development in cell-extrinsic manner. To identify the potential mechanisms, we performed RNA-seq to analyze the gene expression profiles of *Hdac3*-deficient and control MC38 cells, In *Hdac3*-deficient MC38 cells, 810 genes were significantly up-regulated and 519 genes were down-regulated compared to control cells (Fig. 2D). Gene Ontology (GO) enrichment analysis revealed that *Hdac3* deficiency was associated with enriched pathways related to leukocyte migration, neutrophil migration/chemotaxis, cytokine-mediated signaling, and chemokine signaling (Fig. 2E). Gene Set Enrichment Analysis (GSEA) further identified the neutrophil chemotaxis pathway as significantly enriched in *Hdac3*-deficient MC38 cells (Fig. 2F). These findings suggest that *Hdac3* deficiency modulates antitumor immunity by influencing the migration and chemotaxis of various immune cell types within the TIME. Consistent with a previous report [14], we observed elevated levels of CXCL9, CXCL10, and CXCL11, key chemokines involved in CD8^+^T cell chemotaxis, in *Hdac3*-deficient MC38 cells (Fig. 2G). This elevation may represent a critical mechanism underlying the antitumor effects of HDAC3 inhibition. MDSCs, which are pathologically activated neutrophils (PMN-MDSCs) and monocytes (M-MDSCs) with potent immunosuppressive activity, are commonly accumulated in solid tumors, including CRC, where their presence correlates with tumor progression [25]. Notably, our RNA-seq analysis revealed a significant increase in the expression of CXCL1, CXCL2, CXCL3, and CXCL5 in *Hdac3*-deficient MC38 cells. These chemokines are critical ligands that bind to CXCR2, a receptor highly expressed on MDSCs [26–30]. Therefore, the upregulation of CXCL1/2/3/5 may represent a key mechanism that limits the antitumor immune response in response to HDAC3 inhibition. We further validate up-regulation of mRNA levels of CXCL1/2/3/5 in *Hdac3*-deficient MC38 cells using qRT-PCR (Fig. 3A). ELISA also verified an increase in their secretion in the conditioned medium from *Hdac3*-deficient MC38 cells (Fig. 3B). Additionally, up-regulation of HDAC3 mRNA and protein levels was observed in another murine CRC cell line CT-26 (Supplementary Fig. S3A–C). Moreover, we detected elevated HDAC3 mRNA levels in human CRC cell lines HCT-116 and SW-620 following HDAC3 deletion (Fig. 3C and Supplementary Fig. S3D, E). To investigate the impact of HDAC3 inhibition, the specific HDAC3 inhibitor RGFP966 was applied, resulting in increased CXCL1, CXCL2, CXCL3, and CXCL5 levels in both MC38 cells and CRC patient-derived organoids, without affecting organoid viability (Fig. 3D and Supplementary Fig. S3F). Simultaneously, following HDAC3 deficiency or inhibition, the level of the cytotoxic T cell-recruiting chemokine, CXCL10 is also significantly upregulated (Fig. 3E, F). In vitro chemotaxis assays further demonstrated that the supernatant from *Hdac*3-deficient MC38 cells exhibited enhanced ability to promote the migration of both MDSCs and CD8^+^T cells (Fig. 3G, H). Notably, the HDAC3 deficiency-mediated upregulation of MDSC chemotaxis was abrogated in the presence of CXCR2 inhibitor (Fig. 3H). Furthermore, by analysis of the Tumor-Immune System Interaction Database (http://cis.hku.hk/TISIDB/help.php), we found a negative correlation between HDAC3 expression and CD8^+^ T cell infiltration in CRC patients (Fig. 3I). In summary, our data indicates that HDAC3 inhibition enhances recruitment of CD8⁺ T cells via upregulation of CXCL10, and concurrently elevates recruitment of MDSCs by upregulating CXCL1/2/3/5, suggesting a dual role for HDAC3 in regulating anti-tumor immunity in CRC.

### HDAC3 deletion promotes both CD8^+^T cell and MDSC infiltration in syngeneic murine CRC model

**Fig. 4:**
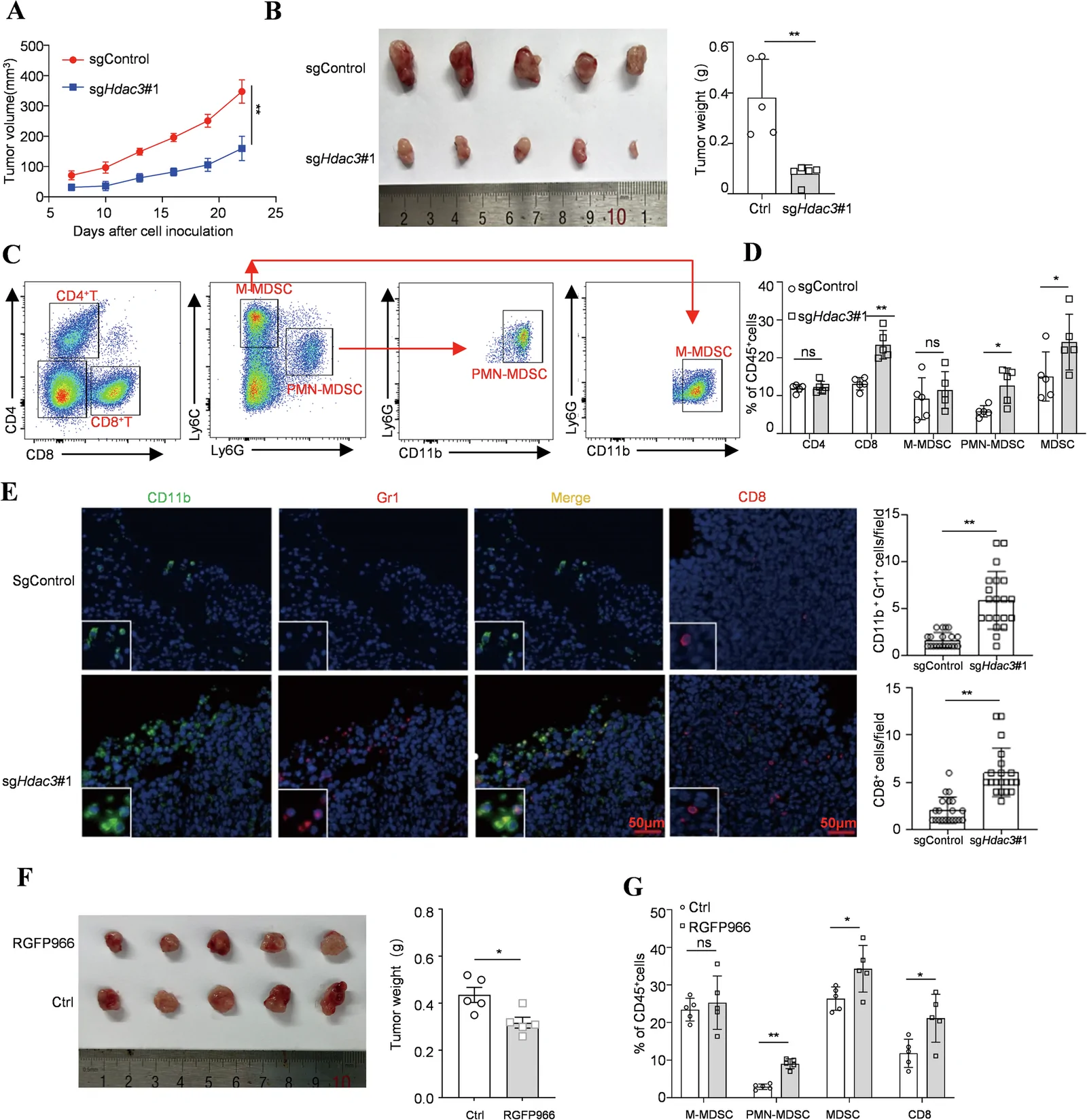
HDAC3 deletion promotes the recruitment of both MDSCs and CD8^+^ T cells in vivo. **A** Tumor growth of mice inoculated s.c. with *Hdac3*-deficient and control MC-38 cells (*n* = 5). **B** Image and tumor weight of *Hdac3*-deficient and control tumor (*n* = 5). **C** Flow cytometric gating strategies for indicated immune cells. **D** Quantification of CD4^+^ T cells, CD8^+^ T cells and MDSCs in *Hdac3*-deficient and control MC38 tumors, evaluated by flow cytometry (*n* = 5). **E** Immunofluorescence analysis and quantifications of infiltration of MDSCs and CD8^+^ T cells in MC38 tumor. **F** Image and tumor weight of MC38 tumor treated with RGFP966 or control. **G** Flow cytometric analysis of CD8^+^ T cells and MDSCs in RGFP966-treated and control MC-38 tumors (*n* = 5). Data are shown as mean ± s.d. (**B**, **D**–**G**) and ± SEM for tumor growth curves (**A**), Statistical significance was evaluated by two-way ANOVA (**A**) or Student’s *t* test (**B**, **D**, **E**). ns not significant; *P < 0.05 and**P < 0.01.

To investigate the role of HDAC3 in tumor growth in vivo, we subcutaneously injected *Hdac3*-deficient and control MC38 cells into C57BL/6 mice. Consistent with previous studies, *Hdac3*-deficient MC38 cells exhibited slower growth compared to control cells in vivo (Fig. 4A, B). As anticipated, flow cytometry analysis revealed increased CD8^+^ T cell infiltration in *Hdac3*-deficient tumors. Interestingly, we also observed elevated infiltration of both total MDSCs and PMN-MDSCs in the TIME of these tumors (Fig. 4C, D). Immunofluorescence analysis further confirmed the increased infiltration of both MDSCs and CD8^+^ T cells in *Hdac3*-deficient tumors (Fig. 4E). Finally, RGFP966 treatment significantly reduced MC-38 tumor growth and increased tumor-infiltrating CD8^+^ T cells (Fig. 4F), accompanied with increased infiltration of CD8^+^ T cells, total MDSCs and PMN-MDSCs (Fig. 4G). Collectively, these results suggest that HDAC3 deletion enhances the recruitment of both CD8^+^ T cells and MDSCs in the TIME of CRC, contributing to the regulation of both anti-tumor and pro-tumor immunity.

### HDAC3 deletion promotes CXCL1/2/3/5/10 expression via an epigenetic mechanism

**Fig. 5:**
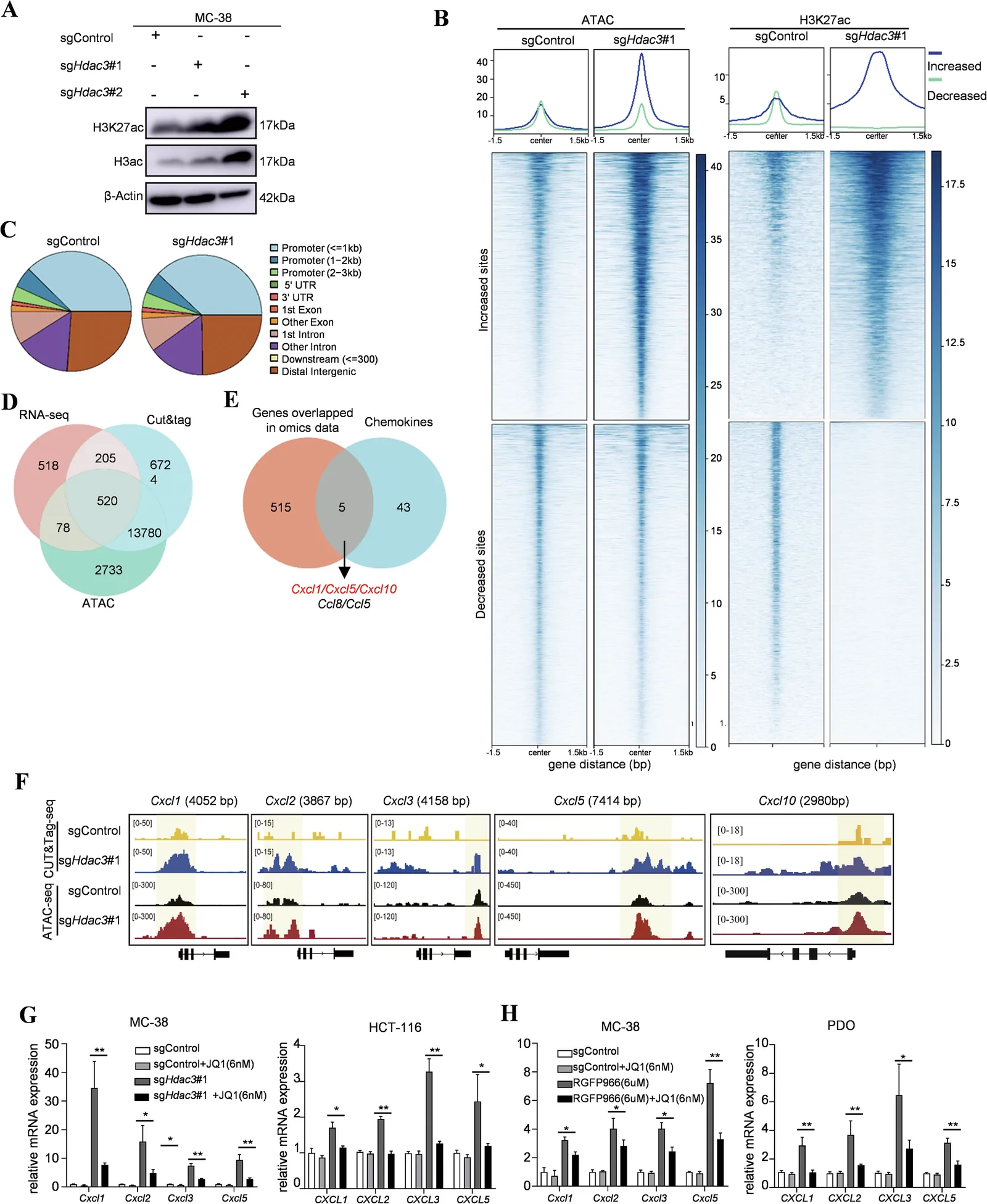
HDAC3 deletion promotes CXCL1/2/3/5 and CXCL10 expression via an epigenetic mechanism. **A** Western blotting analysis of H3K27ac and H3ac expression in *Hdac3*-deficient and control MC38 cells. **B** Heatmap showing ATAC-seq or CUT&Tag-seq coverage at sites with differential chromatin accessibility or altered H3K27ac modification in *Hdac3*-deficient and control MC38 cells. FDR < 0.05. **C** Pie chart indicating the distribution of peaks across the genome in *Hdac3*-deficient and control MC-38 cells using ATAC-seq. **D** Venn diagram demonstrating the overlapped upregulated genes among ATAC-seq, CUT&Tag-seq and RNA-seq in *Hdac3*-deficient MC38 cells. **E** Venn diagram showing overlapped upregulated genes between chemokine gene set and genes enriched in multi-omics data from (**D**). **F** ATAC-seq and H3K27ac CUT&Tag-seq tracks at the genomic regions of CXCL1/2/3/5/10. **G** Relative mRNA levels of *CXCL1/2/3/5* mRNA in *Hdac3*-deficient and control MC-38 cells treated with JQ1 (6 nM). **H** Relative mRNA levels of *CXCL1/2/3/5* in sgControl MC-38 cells or PDOs treated with JQ1 (6 nM) and RGF966 (6 μM) or not (*n* = 3). Data are shown as mean±s.d. of triplicate experiments. Statistical significance was evaluated by Student’s *t* test (**G**, **H**). *P < 0.05 and **P < 0.01.

HDAC3 primarily regulates histone acetylation, particularly at the 27th lysine residue on histone H3 (H3K27), a modification associated with enhanced gene transcription [31]. Western blotting analysis demonstrated that H3K27 was hyperacetylated following HDAC3 deletion (Fig. 5A). Next, we performed H3K27ac-targeted CUT&Tag-seq to identify transcriptionally active genes regulated by HDAC3 in MC38 cells. The results revealed differential transcriptional regulation between *Hdac3*-deficient and control MC-38 cells (Fig. 5B and Supplementary Fig. S4A, B). Additionally, ATAC-seq was used to assess differential chromatin accessibility in MC-38 cells after HDAC3 deletion (Fig. 5B, C), revealing global alterations in chromatin accessibility in *Hdac3*-deficient MC38 cells (Fig. 5B, C). A comprehensive multi-omics analysis integrating RNA-seq, ATAC-seq, and H3K27ac-targeted CUT&Tag sequencing revealed that *Hdac3*-deficient MC-38 cells exhibited the enrichment of 520 genes characterized by elevated mRNA levels, increased chromatin accessibility, and H3K27 hyperacetylation (Fig. 5D). GO analysis indicated that these upregulated genes were significantly associated with cytokine signaling, leukocyte migration, neutrophil migration, and T cell receptor signaling pathways, consistent with previous findings (Supplementary Fig. S4C). Further, we cross-referenced the 520 genes with a known chemokine library [32]and identified five chemokines, with CXCL1, CXCL5, and CXCL10 being the most prominent (Fig. 5E, F).

The bromodomain and extra terminal protein BRD4 directly binds to acetylated lysine on histone tails to facilitate gene transcription by RNA polymerase II [33]. We observed significantly increased BRD4 binding at the promoter region of CXCL1/2/3/5 in *Hdac3*-deficient MC38 cells compared to control cells (Supplementary Fig. S4D). We next asked whether BRD4 was involved in HDAC3 deletion-mediated CXCL1/2/3/5 transcription. JQ1, a BRD4 inhibitor, effectively reversed the upregulation of CXCL1/2/3/5 induced by HDAC3 deletion in both murine and human CRC cells (Fig. 5G, H and Supplementary Fig. S4E, F). These effects appear not to be toxic to JQ1 at this dose in MC38 cells (Supplementary Fig. S5A–C). Collectively, these results suggest that HDAC3 deficiency enhances chromatin accessibility and H3K27 hyperacetylation, leading to active transcription of CXCL1/2/3/5/10.

### CXCR2 blockade further suppress *Hdac3*-deficient tumor growth by inhibiting the recruitment of MDSCs

**Fig. 6:**
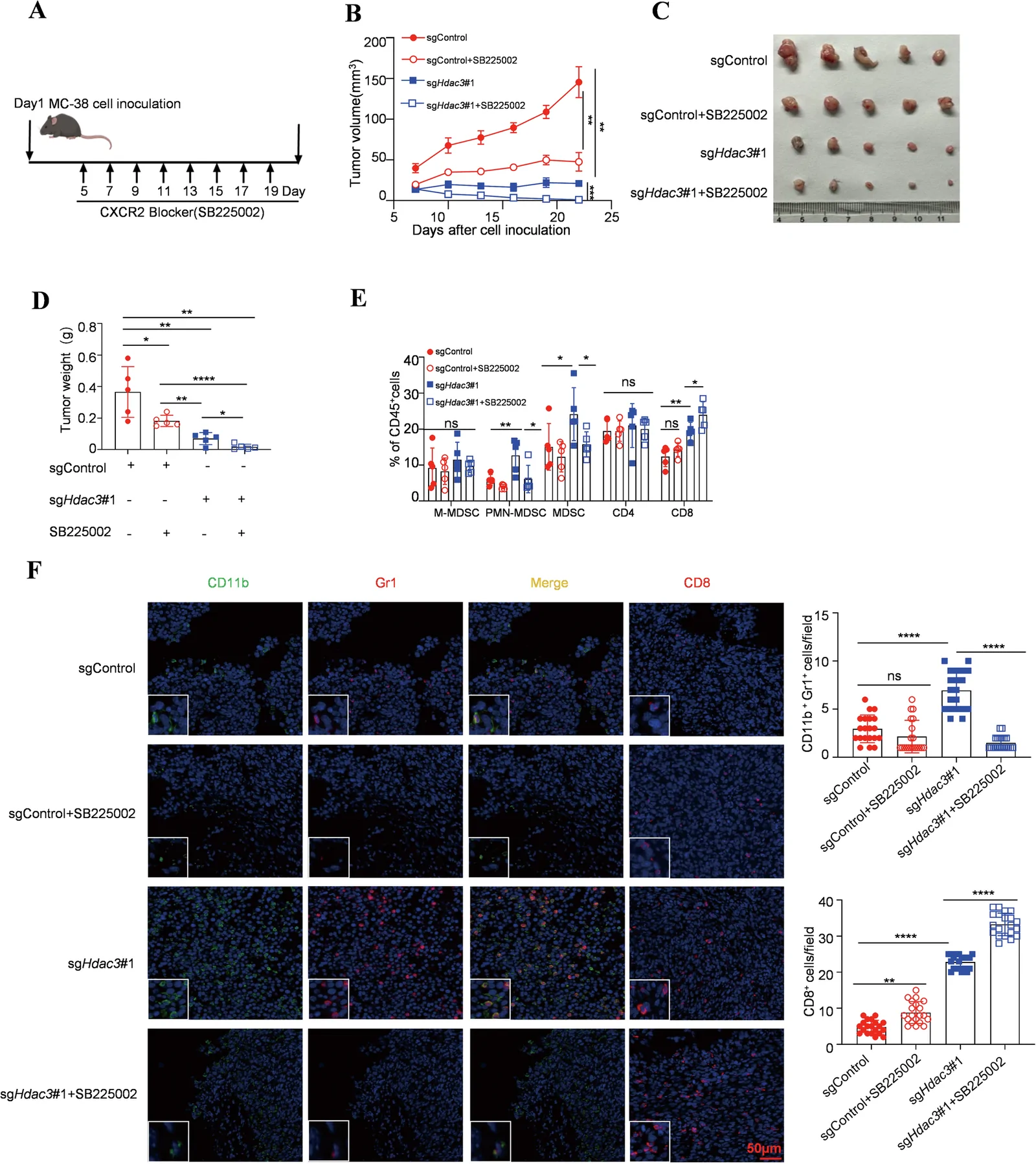
CXCR2 blockade synergizes with HDAC3 inhibition to suppress tumor growth. **A** Schematic flow diagram of the experiment design. **B**–**D** The effects of SB225002 on tumor growth of mice inoculated s.c. with *Hdac3*-deficient and control MC38 cells (*n* = 5). **E** Quantifications of immune cell infiltration in tumors, determined by flow cytometry. **F** Immunofluorescence analysis and quantifications of infiltration of MDSCs and CD8^+^ T cells in indicated MC38 tumor. Data are shown as mean ± s.d. (**D**–**F**) and ±s.m. (**B**). Statistical significance was evaluated by two-way ANOVA (**B**) or Student’s *t* test (**D**–**F**). ns not significant; *P < 0.05, **P < 0.01, ***P < 0.001 and ****P < 0.0001.

We next asked whether increased infiltration of MDSCs could compromise HDAC3 deletion-mediated antitumor immunity. To this aim, we treated mice bearing MC38 tumors with CXCR2 inhibitor (SB225002) (Fig. 6A), which has been proved to inhibit the recruitment of MDSCs in the TIME [26]. As anticipated, SB225002 treatment further suppressed *Hdac3*-deficient tumor growth, and in some cases, leading to complete tumor regression (Fig. 6B–D). Flow cytometry analysis revealed that SB225002 treatment effectively reduced the infiltration of both total MDSCs and PMN-MDSCs in *Hdac3*-deficient tumors, while simultaneously enhancing CD8^+^ T cell infiltration (Fig. 6E). Immunofluorescence analysis further confirmed the altered distribution of MDSCs and CD8^+^ T cells within the tumors (Fig. 6F). These findings provide evidence that HDAC3 inhibition promotes the recruitment of MDSCs to limit its anti-tumor immunity, blocking the recruitment of MDSCs enables enhanced efficacy of HDAC3 inhibition therapy for colorectal cancer.

## Discussion

HDAC3 has been shown to be upregulated in human CRC and is implicated in the carcinogenesis of CRC [20, 34]. However, the role of HDAC3 in CRC remains controversial. Consistent with a recent report [14], our findings indicate that HDAC3 inhibition does not affect CRC cell survival or proliferation, despite the cytotoxic effects on multiple other human cancer types [35, 36]. In contrast, HDAC3 inhibition enhances antitumor immunity in a murine CRC model. These results underscore the significant role of HDAC3 in promoting CRC progression through immune-related mechanisms.

A previous study demonstrated that HDAC3 inhibition promotes the recruitment of CD8^+^T cells into the TIME in a murine fibrosarcoma model by upregulating CXCL9/10/11 [14]. Consistent with these findings, we also observed upregulation of CXCL9/10/11 in several murine and human CRC cell lines, as well as in human CRC organoids. Additionally, we detected increased infiltration of CD8^+^T cells into the TIME in the murine CRC model. Given the critical role of CD8^+^ T cells in antitumor immunity, these findings suggest that HDAC3 inhibition-induced CD8^+^ T cell infiltration is a key mechanism underlying its antitumor effects.

Surprisingly, both HDAC3 deficiency and inhibition significantly increase the expression of chemokines CXCL1, CXCL2, CXCL3 and CXCL5 in several murine and human CRC cell lines. These chemokines serve as critical ligands for the chemokine receptor CXCR2, which is highly expressed on MDSCs [37]. Indeed, the supernatant from *Hdac3*-deficient MC38 cells exhibit an enhanced ability to promote MDSC migration. This effect was completely abrogated by a specific CXCR2 inhibitor, suggesting that HDAC3 deficiency facilitates MDSC migration via the upregulation of the CXCL1/2/3/5- CXCR2 signal axis. Further analysis revealed that while HDAC3 deletion slowed tumor growth in a murine CRC model, the tumors contained increased number of MDSCs within the TIME alongside increased presence of CD8^+^ T cells. Notably, a combination of HDAC3 deficiency and CXCR2 inhibition synergistically suppressed CRC growth in immunocompetent mice, highlighting that HDAC3 deletion-induced MDSC infiltration into the TIME compromises antitumor immunity. Moreover, HDAC3 expression levels inversely correlate with the infiltration of both CD8^+^ T cells and MDSCs in human CRC specimens. These findings suggest that HDAC3 inhibition promotes the recruitment of both CD8^+^T cells and MDSCs in CRC, thereby precisely regulating anti-tumor immunity. Owing to the inherent technical limitations of scRNA-seq platforms in capturing PMN-MDSCs, a correlation analysis between HDAC3 expression and CXCR2⁺ MDSC infiltration in human CRC was not performed in this study. However, future validation in independent cohorts using immunohistochemistry or flow cytometry is warranted. The increased chemotaxis and recruitment of MDSCs to the TIME appears to be a key mechanism that limits antitumor immunity [38]. HDAC3 inhibition has been reported to upregulate co-inhibitory molecules, such as PD-L1, in certain tumor cells, thereby potentially antagonizing antitumor immunity [9, 39]. Moreover, we observed that HDAC3 inhibition upregulated the expression of CCL2, CCL5 and CCL8 in MC38 cells, suggesting a potential role in modulating the infiltration of additional immune cell populations beyond CD8⁺ T cells and MDSCs. Collectively, these findings indicate that HDAC3 inhibition regulates tumor immunity through multiple mechanisms.

HDAC3 deletion induces a rapid increase in both H3K27 acetylation and chromatin accessibility at the genomic regions of CXCL1, CXCL2, CXCL3, CXCL5 and CXCL10. Consistent with this, treatment with the BRD4 inhibitor dramatically downregulated the expression of CXCL1, CXCL2, CXCL3 and CXCL5 in HDAC3-deficient CRC cells, providing an important mechanism by which HDAC3 inhibition upregulates the expression of these chemokines. HDAC3 primarily acts as an epigenetic regulator that does not directly bind chromatin to deacetylate histone tails. Further studies are required to identify the transcription factors that facilitate the recruitment of HDAC3 to the genomic regions of CXCL1/2/3/5/10.

HDAC3 inhibition has been reported to promote antitumor immunity through the recruitment of CD8⁺ T cells [14]. Here we find that HDAC3 inhibition also suppresses antitumor immunity by promoting MDSC migration via upregulation of CXCL1, CXCL2, CXCL3 and CXCL5. Thus, this study reveals a previously unappreciated dual role for HDAC3 inhibition in tumor immune responses. These findings may help to explain the limited efficacy of single-agent HDAC3 inhibition in clinical trials [24] and supports a novel therapeutic strategy combining CXCR2 blockade. However, this study has certain limitations: we primarily focused on the quantity and chemotaxis of MDSCs but did not thoroughly investigate whether HDAC3 deficiency recruits MDSCs with stronger immunosuppressive functions, the characterization of MDSC functional subsets and their immunosuppressive capabilities remain insufficient. In the complex tumor microenvironment in vivo, other immune cells or stromal cells might feedback-regulate the chemokine expression in tumor cells through cytokine secretion, thereby forming a regulatory circuit. These aspects warrant further investigation in future studies.

**Fig. 7:**
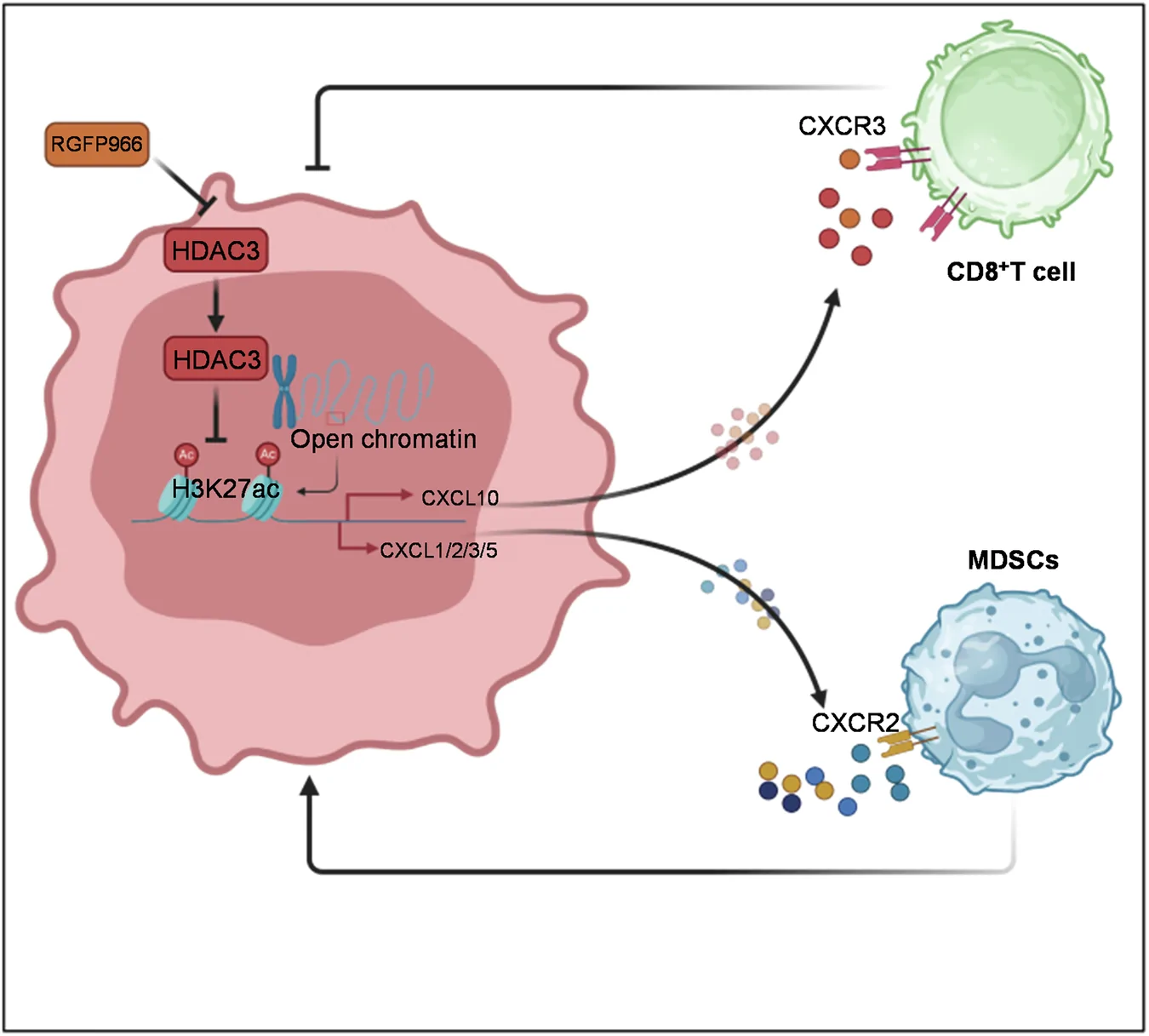
A schematic representation of the dual roles mediated by HDAC3 inhibition in regulating tumor immunity. HDAC3 inhibition enhances chromatin accessibility and augments histone H3K27 acetylation to up-regulate CXCL10 and CXCL1/2/3/5 expression. CXCL10 up-regulation recruits CD8⁺ T cells via CXCR3 to reinforce anti-tumor immunity. Meanwhile, CXCL1/2/3/5 up-regulation recruits MDSCs act via CXCR2 to blunt anti-tumor immune responses.

Our study demonstrates that HDAC3 inhibition promotes the recruitment of MDSCs into the TIME, thereby impairing antitumor immunity. Combined use of a CXCR2 inhibitor can effectively counteract HDAC3-mediated immunosuppression and significantly enhance therapeutic efficacy (Fig. 7). Providing novel targets and strategies for overcoming immunotherapy resistance and developing combination therapies in CRC.

## Supplementary information

uncropped western blot

Supp figure

supp table

## Data Availability

Data sets generated during the current study are available from the corresponding author on request.
