# Supplementary material for: CXCR2 blockade potentiates HDAC3 deletion mediated antitumor immunity in colorectal cancer
Source: Cell Death Dis. 2026 Jul 10;17(1):862. doi: 10.1038/s41419-026-09006-3 (PMC13639080; doi:10.1038/s41419-026-09006-3)

Original data for Figure 1C

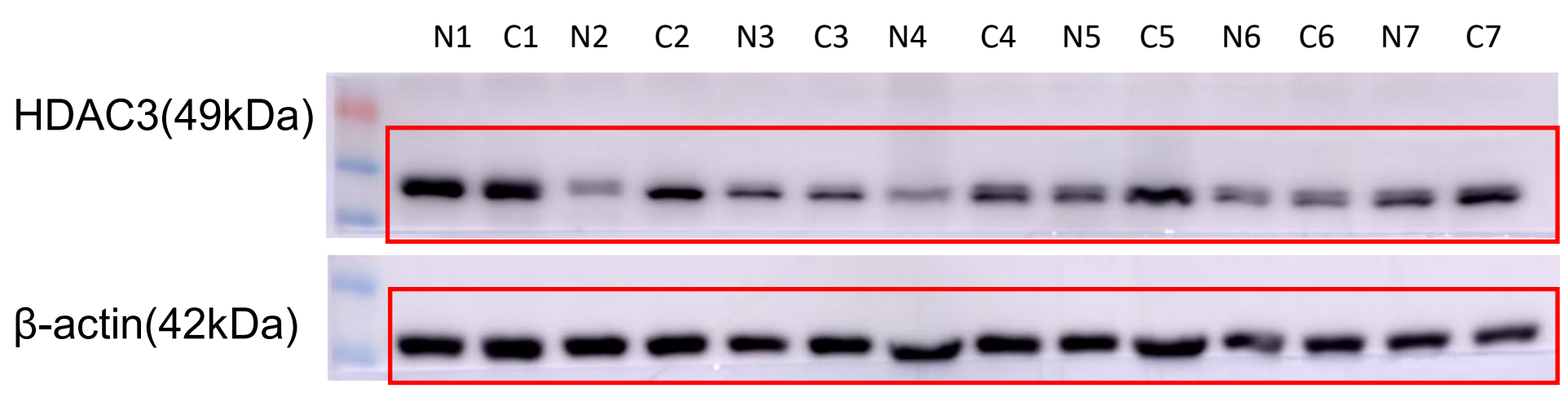

Original data for Fiure 2A

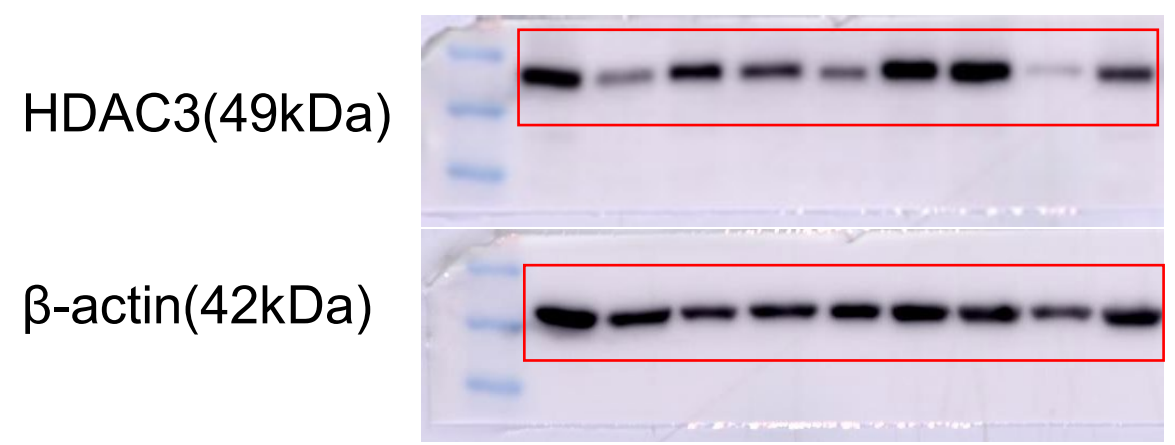

Original data for Figure 2B

Original data for Figure S3E

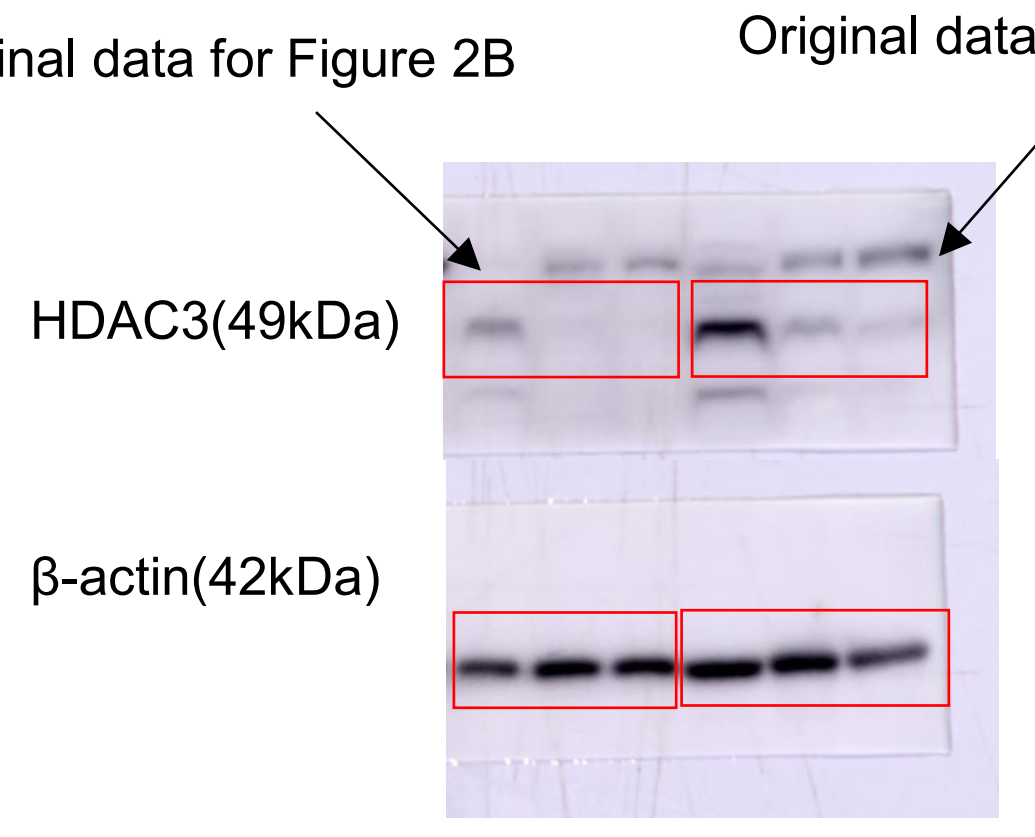

Original data for Figure 5A

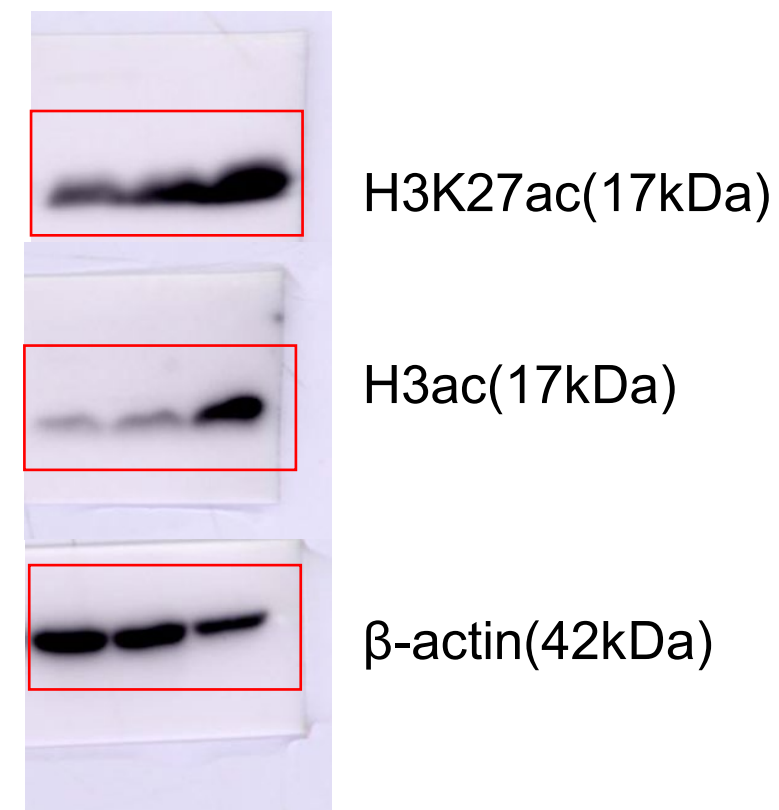

Original data for Figure S3A

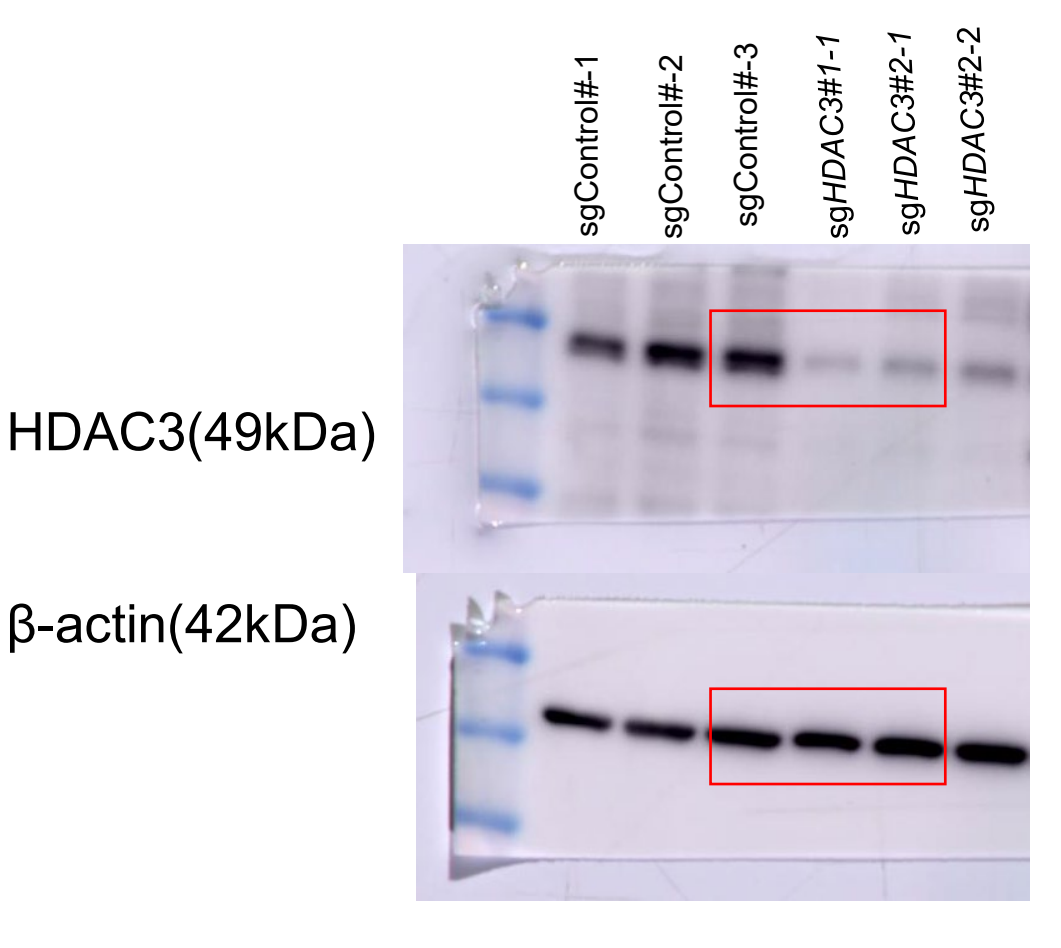

Original data for Figure S3D

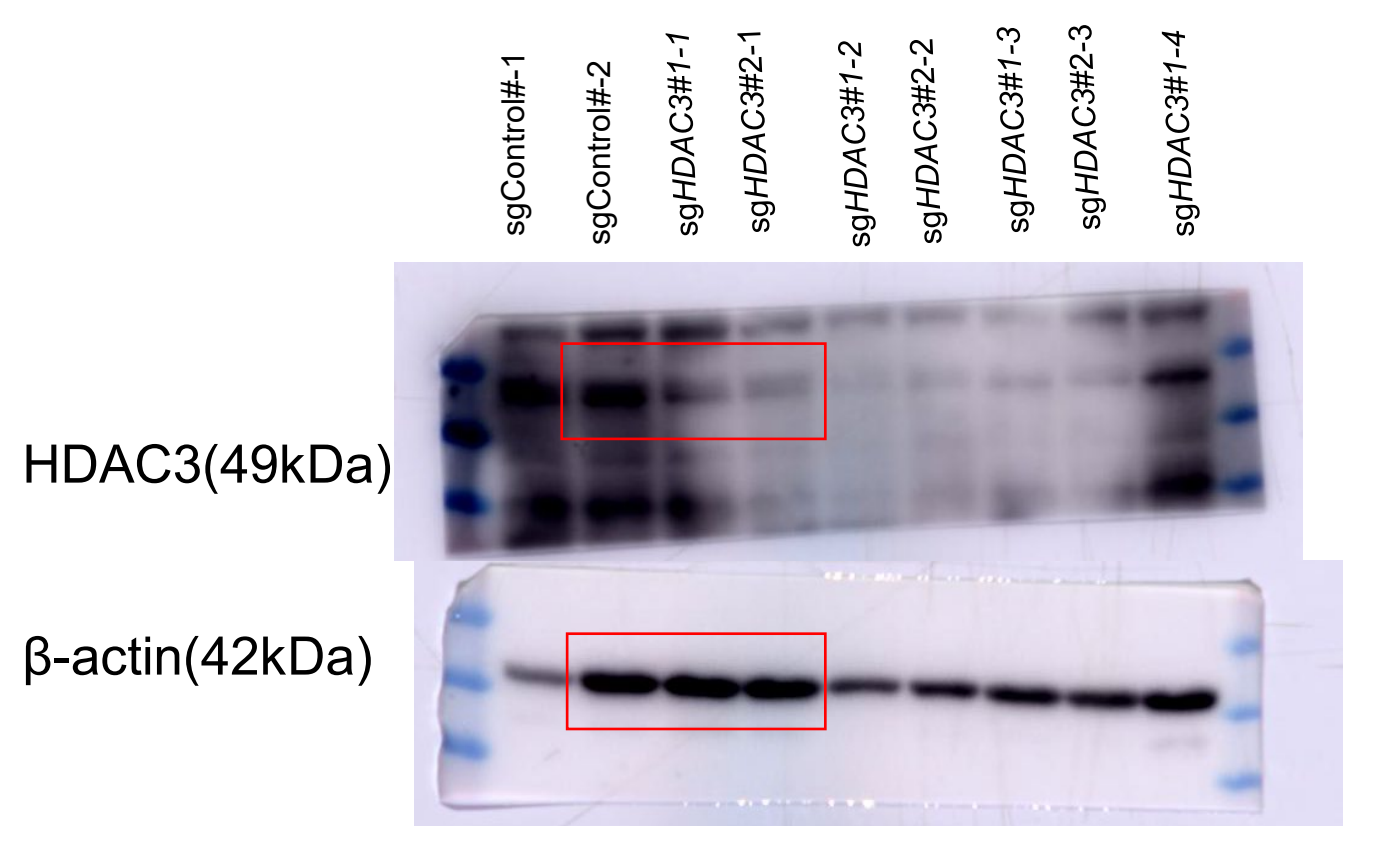

Supplement: Supplementary file 1 [file 41419_2026_9006_MOESM1_ESM.pdf]
