# Supplementary material for: CXCR2 blockade potentiates HDAC3 deletion mediated antitumor immunity in colorectal cancer
Source: Cell Death Dis. 2026 Jul 10;17(1):862. doi: 10.1038/s41419-026-09006-3 (PMC13639080; doi:10.1038/s41419-026-09006-3)
Supplement: Supplementary file 2 [file 41419_2026_9006_MOESM2_ESM.pdf]

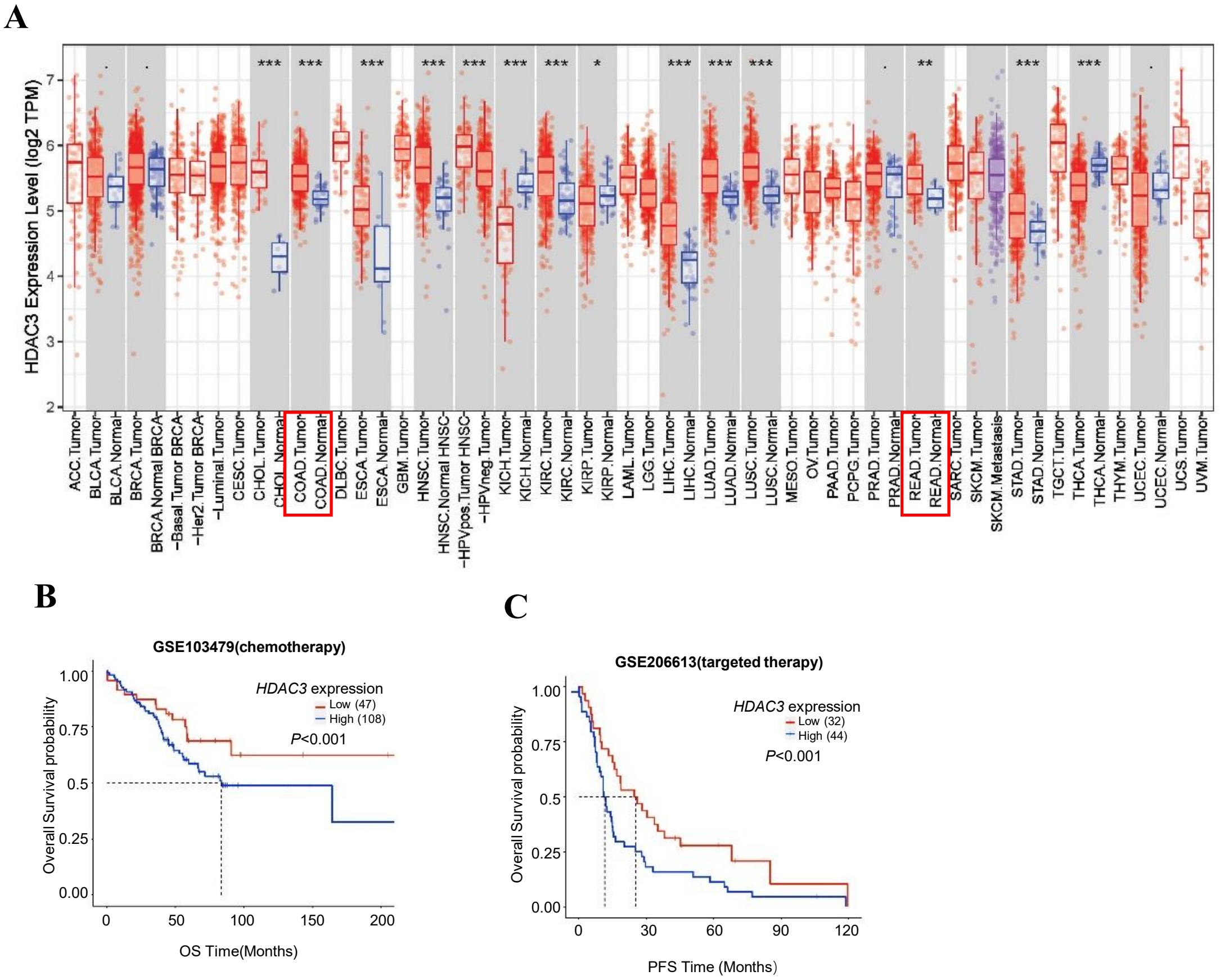

Figure S1. HDAC3 expression is upregulated in various tumors and predicts worse outcome in CRC treatment. A, The expression levels of HDAC3 mRNA in different types of human tumors and matched normal tissues in The Cancer Genome Atlas (TCGA) (TIMER2.0, http://timer.cistrome.org/). Wilcoxon test was used to evaluate the statistical significance. **B**, Kaplan-Meier analysis of overall survival of CRC patients based on *HDAC3* expression after chemotherapy (GSE103479). **C**, Kaplan-Meier analysis of Progression-Free-Survival of CRC patients based on *HDAC3* expression after targeted therapy (GSE206613). **P*<0.05, ***P*<0.01, ****P*<0.001.

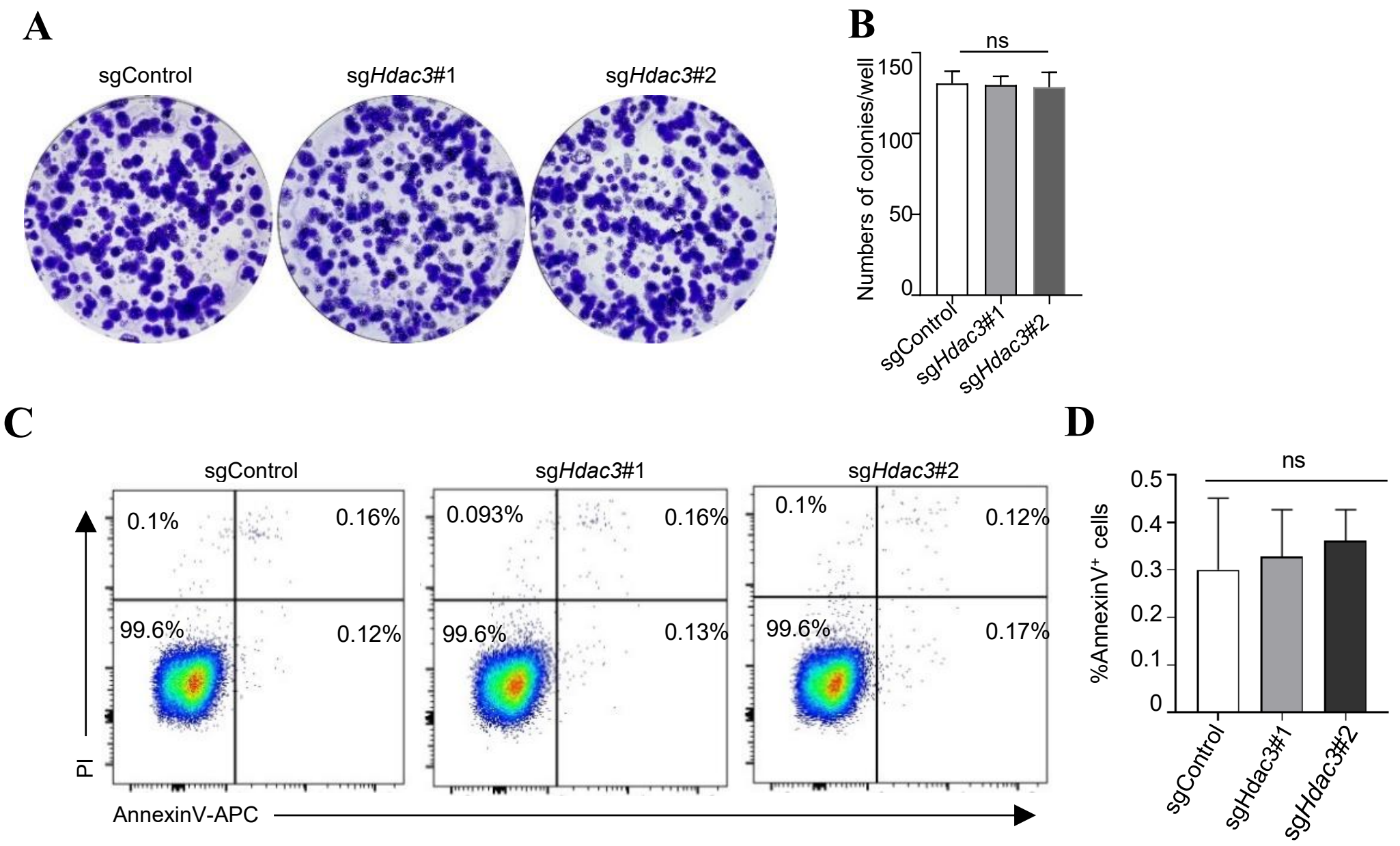

Figure S2. **HDAC3 knockout did not affect the proliferation and apoptosis in CRC. A-B**, Colon formation of the *Hdac3* knockout and control MC-38 cells. **C-D**, Apoptosis analysis of the *Hdac3* knockout and control MC-38 cells. Statistical significance was evaluated by Student's t-test (**B, D**). ns, not significant.

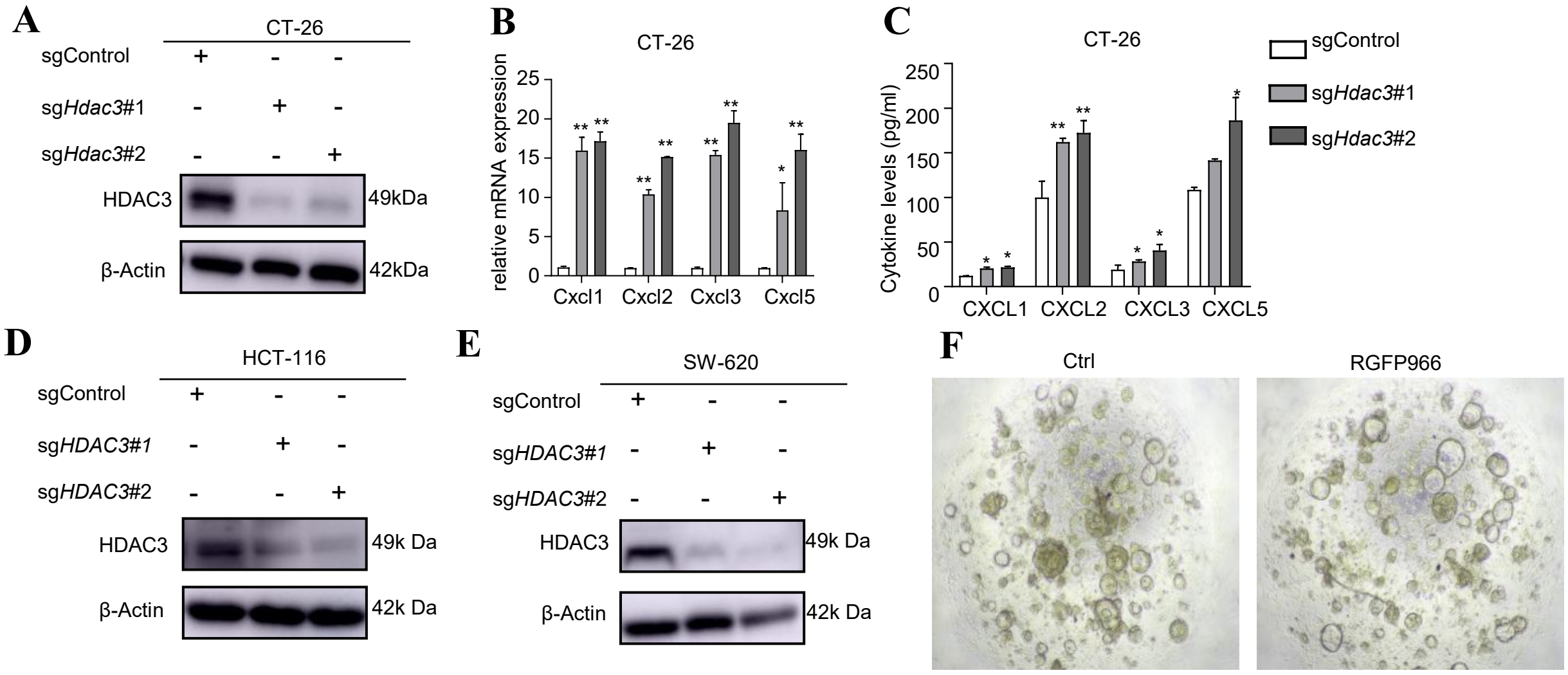

Figure S3. HDAC3 knockout or inhibition upregulated the CXCL1/2/3/5 expression. **A**, Western blot confirmed the knockout of *Hdac3* in CT-26 cells. **B**, RT-qPCR validated that *Hdac3* knockout upregulated CXCL1/2/3/5 mRNA in CT-26 cells (n=3). **C**, ELISA confirmed HDAC3 knockout upregulated CXCL1/2/3/5 secretion in CT-26 cells (n=3). **D-E**, Western blot confirmed the knockout of *HDAC3* in HCT-116 and SW-620 cells. **F**, Representative images of the CRC patient-derived organoids after treatment of RGFP966. Data are shown as mean ± s.d. (**B and C**). Statistical significance was evaluated by Student's t-test (**B and C**). *$P<0.05$ and **$P<0.01$.

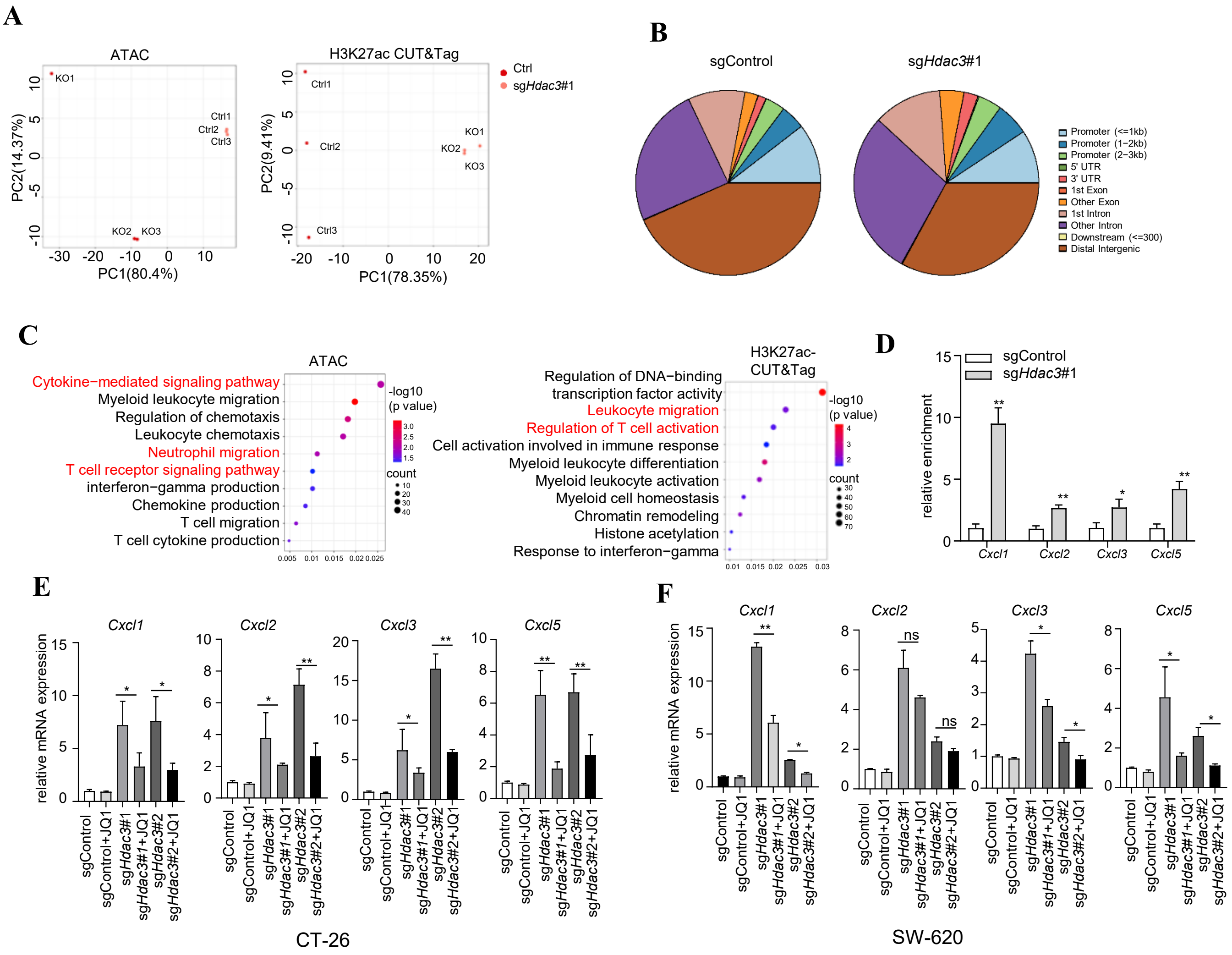

Figure S4. HDAC3-KO upregulates CXCL1/2/3/5 by regulating the chromatin open extent and histone acetylation. A, PCA of ATAC and CUT&Tag sequence in *Hdac3* knockout and control MC-38 cells (n=3). **B**, Pie chart indicates the distribution of peaks across the genome in *Hdac3* knockout and control MC-38 cells in H3K27ac CUT&Tag-seq (n=3). **C**, GO pathway enrichment analysis of upregulated genes (FDR<0.05) in *Hdac3* knockout MC-38 cells compared to control group (n=3). **D**, Cut&Tag-QPCR analysis of the relative enrichment of BRD4 at indicated genomic sites (n=3). **E-F**, Relative expression of CXCL1/2/3/5 mRNAs in HDAC3 deficient or control CT-26 cells and SW-620 cells treated with JQ1 or not (n=3). Data are shown as mean ± s.d. (**D-F**). Statistical significance was evaluated by Student's t-test (**D-F**). ns, not significant; **P*<0.05 and ***P*<0.01.

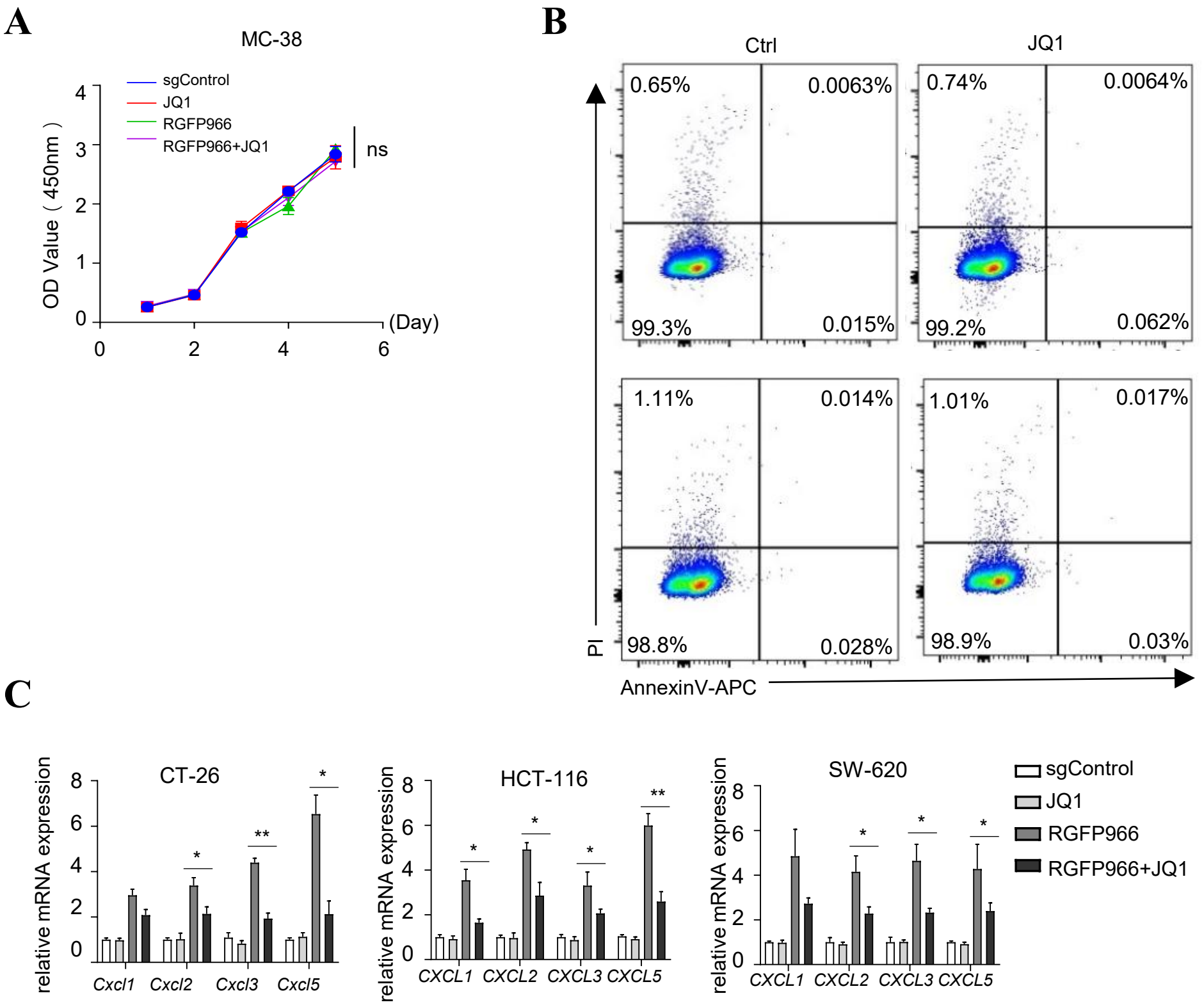

Figure S5. HDAC3 inhibition induced CXCL1/2/3/5 upregulation could be rescued by JQ1 . A-B, The effect of RGFP966 and JQ1 on the proliferation and apoptosis of MC-38 cells (n=3). **C**, Relative expression of CXCL1/2/3/5 mRNAs in sgControl CT-26 cells, HCT-116 and SW-620 cells treated with JQ1 or RGFP966 (n=3). Data are shown as mean ± s.d. (**C**). Statistical significance was evaluated by Student's t-test (**C**) and two-tailed two-way ANOVA (**A**). ns, not significant; **P*<0.05 and ***P*<0.01.
